# Extremely Thermostabilizing Core Mutations in Coiled-Coil Mimetic Proteins of HIV-1 gp41 Produce Diverse Effects on Target Binding but Do Not Affect Their Inhibitory Activity

**DOI:** 10.3390/biom11040566

**Published:** 2021-04-12

**Authors:** Mario Cano-Muñoz, Samuele Cesaro, Bertrand Morel, Julie Lucas, Christiane Moog, Francisco Conejero-Lara

**Affiliations:** 1 Departamento de Química Física, Instituto de Biotecnología, Unidad de Excelencia de Química Aplicada a Biomedicina y Medioambiente (UEQ), Facultad de Ciencias, Universidad de Granada, 18071 Granada, Spain; samuele.cesaro@univr.it (S.C.); bmorel@ugr.es (B.M.); 2 INSERM U1109, Fédération de Médecine Translationnelle de Strasbourg (FMTS), Université de Strasbourg, 67084 Strasbourg, France; julie.lucas@etu.unistra.fr (J.L.); c.moog@unistra.fr (C.M.)

**Keywords:** HIV, AIDS, envelope glycoprotein, stability, fusion inhibitors, calorimetry, peptides, binding

## Abstract

A promising strategy to neutralize HIV-1 is to target the gp41 spike subunit to block membrane fusion with the cell. We previously designed a series of single-chain proteins (named covNHR) that mimic the trimeric coiled-coil structure of the gp41 N-terminal heptad repeat (NHR) region and potently inhibit HIV-1 cell infection by avidly binding the complementary C-terminal heptad repeat (CHR) region. These proteins constitute excellent tools to understand the structural and thermodynamic features of this therapeutically important interaction. Gp41, as with many coiled-coil proteins, contains in core positions of the NHR trimer several highly conserved, buried polar residues, the role of which in gp41 structure and function is unclear. Here we produced three covNHR mutants by substituting each triad of polar residues for the canonical isoleucine. The mutants preserve their helical structure and show an extremely increased thermal stability. However, increased hydrophobicity enhances their self-association. Calorimetric analyses show a marked influence of mutations on the binding thermodynamics of CHR-derived peptides. The mutations do not affect however the in vitro HIV-1 inhibitory activity of the proteins. The results support a role of buried core polar residues in maintaining structural uniqueness and promoting an energetic coupling between conformational stability and NHR–CHR binding.

## 1. Introduction

HIV-1 infection remains a global pandemic with more than 38 million of people living with the virus and near 1.7 million new infections in 2019 [1]. Despite current efforts to develop active and passive immunization strategies [2], no effective vaccines have been approved. Highly active antiretroviral therapies (HAART) have improved considerably the life expectancy of infected patients, but the emergence of multidrug-resistant viral strains [3] and the appearance of adverse effects and drug–drug interactions in some patients [4] highlight the need for new improved antivirals and microbicides to combat the infection.

Cell infection by HIV-1 is mediated by its envelope (Env) glycoprotein [5]. Env is a trimer of heterodimers with three external gp120 and three transmembrane gp41 subunits [6]. Viral attachment to cells is initiated by gp120, which binds to the CD4 cell receptor and a co-receptor. This event triggers gp120 shedding from gp41 and a large conformational change in the latter that inserts its N-terminal fusion peptide in the cell membrane and forms an extended intermediate that bridges the cell and viral membranes [7]. Subsequently, gp41 folds onto itself forming a highly stable six-helix-bundle (6HB), composed of a central parallel trimeric coiled coil formed by the N-terminal heptad repeat region (NHR) surrounded by three antiparallel C-terminal heptad repeat (CHR) helices [8,9]. This energetically favorable folding process brings the viral and the cell membranes into close proximity facilitating their fusion and thereby the insertion of the viral material into the cell.

Blocking Env-mediated cell-virus membrane fusion has been considered for more than two decades a promising approach to inhibit HIV infection [5,10,11]. Due to its key role in the fusion process, the gp41 extended intermediate is an attractive target and has become a subject of intense research. In this intermediate, both the NHR and CHR regions of gp41 are exposed and can be accessed by a variety of molecules, including complementary peptides derived from NHR or CHR regions, artificial D-peptides, antibodies, and small-molecule compounds [12,13,14,15,16]. The only HIV-1 inhibitor of this type in clinical use is T20, known by its generic name as enfuvirtide [17], but its use is strongly limited by its low efficacy, short half-life, rapid acquisition of drug resistance, and high cost. Other peptides and small-molecule compounds directed against the coiled-coil structure of NHR have been developed, although very few have reached clinical stage [18].

Compared to CHR peptides, NHR-based peptide mimetics have a generally lower anti-HIV-1 activity due to their low solubility and tendency to aggregate. These problems can be alleviated by engineered protein constructs that imitate exposed NHR trimers. These constructs have shown potent inhibition activities and have certain advantages such as their activity against strains resistant to CHR inhibitors [10,19,20,21].

Despite all these advances, the molecular and energetic determinants of the NHR–CHR interaction are still not fully understood, making it difficult to improve existing inhibitors and develop new molecules, in particular small molecule drugs, targeting gp41.

We previously designed a series of protein molecules, called covNHR, that structurally mimic the trimer NHR helices of gp41 [22]. These proteins consist of single polypeptide chains that fold in a helix–loop–helix–loop–helix topology as antiparallel coiled-coil trimers. These covNHR proteins are very stable, bind CHR-derived gp41 peptides with high affinity, and inhibit infection through a broad variety of HIV-1 pseudoviruses and primary isolates. The crystallographic high-resolution structure of the complex between a covNHR variant and the CHR peptide C34 [23] shows a remarkable similarity with the CHR–NHR binding interface of the post-fusion structure of gp41 [9], including the presence of buried interfacial water molecules. Outstandingly, the change in the coiled-coil topology from parallel to antiparallel of the NHR trimer of helices is very well tolerated, and there is a remarkable preservation of core-packing inter-helical contacts, despite the different spatial orientation of the Cα-Cβ bond in the side chains of the antiparallel helix relative to the parallel ones.

Given that our covNHR proteins are highly soluble and stable, easy to produce in sufficient quantities, and bind the CHR peptides with 1:1 stoichiometry, they are particularly amenable for thermodynamic studies of the CHR–NHR interaction. We have recently made a thermodynamic dissection of this interaction and found that the binding energy is distributed among several binding pockets and that there is allosteric communication between them [24].

An interesting feature of the coiled coils in many proteins is the presence of non-canonical polar residues in a and d core positions of the heptad repeats, which has been related to specificity and structural uniqueness at the expense of stability [25]. The NHR coiled-coil region of gp41 contains three of such buried polar residues, namely Q41, Q51, and T58 (Q562, Q572, and T569 in full Env sequence numbering). These residues occupy positions a, d, and d in their respective heptad repeats, and their polar side chains mutually satisfy part of their hydrogen bonding potential often involving buried water molecules and backbone carbonyl oxygen atoms. In the case of the covNHR protein, the buried polar residues are Q13, Q99, and Q124 (structurally equivalent to Q41); Q23, Q89, and Q134 (equivalent to Q51); and T30, T82, and T141 (equivalent to T58). In our published structure (Protein Data Bank (PDB) id. 6R2G), a water molecule occupies a small cavity between the side chains of the Q23, Q89, and Q134 triad. Although these hydrogen bonds are expected to be stronger in the apolar environment of the coiled-coil core [26], their number is smaller than in a fully hydrated environment. Moreover, there is a considerable entropy penalty due to the geometric constraints imposed by the formation of these hydrogen bonds and in the immobilization of the buried water molecules. In contrast, canonical hydrophobic residues in trimeric coiled coils (Ile, Val, Leu) are expected to be energetically favored in core positions. This was demonstrated in a study of an NHR(L6)CHR construct of the simian immunodeficiency virus (SIV) gp41 core [27], in which the polar core residues were individually mutated to isoleucine. The authors found that core polar-to-hydrophobic mutations have strongly stabilizing effects. However, since the constructs used were mimics of the post-fusion 6HB conformation, the energetic consequences of these core mutations on the CHR–NHR interaction cannot be separated from their effects on the central NHR coil stability.

**Figure 1:**
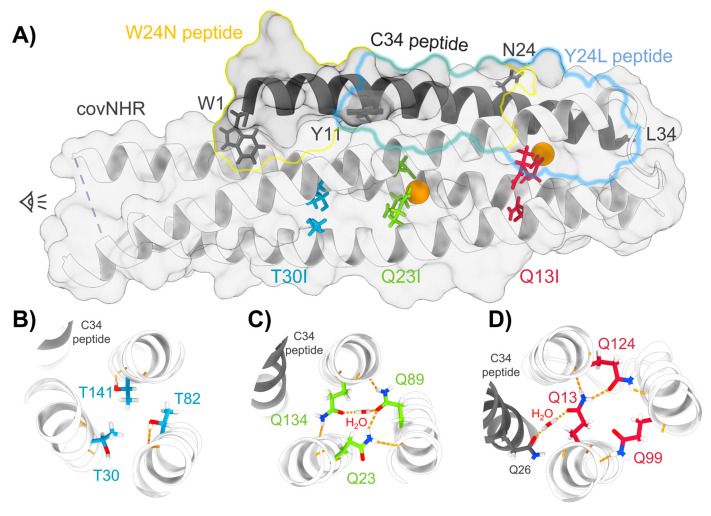
Buried polar amino acids in the coiled-coil structure of covNHR. (**A**) Representation of the X-ray structure of the complex of covNHR with W34L (C34) (PDB id. 6R2G) [23]. The limits of the W24N and Y24L peptides are highlighted on the structure with yellow and blue lines, respectively. The buried polar side chains are represented with sticks and buried water molecules with orange spheres. (**B**–**D**) Cross-sectional representations of each triad of polar side chains showing hydrogen bonds in dashed yellow lines.

We hypothesized that buried polar residues may be involved in the mechanisms by which the NHR–CHR interaction is regulated. Since the covNHR proteins constitute an excellent model to investigate the molecular and energetic details of this interaction, we produced three triple mutants (Figure 1), in which each triad of polar residues was replaced by isoleucine. We selected isoleucine over leucine because trimeric coiled coils can accommodate beta-branched amino acids at both a and d positions of the heptad repeat due to their acute knobs-into-holes packing, whereas leucine is favored at positions with perpendicular packing present in other helical arrangements [28]. For the sake of simplicity, each mutant was named after the first sequence position mutated. We investigated the stability of the three variants in comparison to the reference protein and their capacity to recognize their CHR target sequence, as well as their inhibitory activity against in vitro HIV-1 infection. The results indicate significant influence of the core mutations upon the CHR–NHR interaction.

## 2. Materials and Methods

### 2.1. Proteins and Peptides

The DNA encoding protein sequences were synthesized and cloned into pET303 expression vectors by Thermo Fisher Scientific (Waltham, Massachusetts, MA, USA). To facilitate purification by Ni-Sepharose affinity chromatography, the protein sequences included a C-terminal histidine tag with sequence GGGGSHHHHHH. The sequences are shown in Table S1. Each triple mutant was named as covNHR followed by the identity of first mutation, i.e., covNHR-Q13I, covNHRQ23I, and covNHR-T30I. The proteins were overexpressed in *E. coli* and purified, as described previously [22]. The protein purity was assessed by SDS-PAGE, and the identity of each protein variant was confirmed by mass spectrometry analysis.

Synthetic CHR peptides (Table S1), both N-acetylated and C-amidated, were acquired from Genecust (Mondorf-les-Bains, Luxembourg), with a purity >95%. Protein and peptide concentrations were measured by UV absorption measurements at 280 nm with extinction coefficients calculated according to their respective amino acid sequences with the ExPasy ProtParam server (https://web.expasy.org/protparam/, accessed on 3 March 2020) [29].

### 2.2. Circular Dichroism

Circular dichroism (CD) measurements were carried out in a Jasco J-715 spectropolarimeter (Jasco, Tokyo, Japan) equipped with a Peltier thermostatic cell holder. Measurements of the far-UV CD spectra (260–200 nm) were made with a 1 mm path-length quartz cuvette at a protein concentration of ~15 μM. Buffer conditions were 50 mM sodium phosphate pH 7.4. Spectra were obtained by averaging 5 scans recorded at a scan rate of 100 nm/min, 1 nm step resolution, 1 s response, and 1 nm bandwidth. Thermal unfolding was monitored by measuring the CD signal at 222 nm as a function of temperature using a scan rate of 1 °C min^−1^.

### 2.3. Light Scattering

The apparent hydrodynamic radii of the proteins were measured using dynamic light scattering (DLS) in a DynaPro MS-X DLS instrument (Wyatt, Santa Barbara, CA, USA). Dynamics v6 software (Wyatt Technology Corporation, Santa Barbara, CA, USA) was used in data collection and processing. Sets of DLS data were measured at 25 °C with an average number of 50 acquisitions and an acquisition time of 10 s. Measurements were carried out in 50 mM sodium phosphate buffer pH 7.4 and 50 mM glycine/HCl buffer pH 2.5.

Static scattering intensities were measured in a Malvern μV instrument (Malvern Panalytical, Malvern, UK) at 25 °C, in 50 mM sodium phosphate buffer pH 7.4, at different concentrations of protein in a range of 0.2 to 3.5 mg mL^−1^. The intensities were analyzed using the Debye plot as represented by Equation (1),

K_C_/R_90_ = 1/M_W_ + 2A_2_c (1)

valid for particles significantly smaller than the wavelength of the incident radiation, where the *K* is an optical constant of the instrument, *c* is the particle mass concentration, *R*_90_ is the Rayleigh ratio of scattered to incident light intensity, *M_w_* is the weight-averaged molar mass, and *A*_2_ is the 2nd virial coefficient that is representative of inter-particle interaction strength. *M_w_* can be determined from the intercept of the plot.

### 2.4. Differential Scanning Calorimetry

Differential scanning calorimetry (DSC) experiments were carried out in a MicroCal PEAQ-DSC microcalorimeter equipped with an autosampler (Malvern Panalytical, Malvern, UK). Scans were run from 5 to 130 °C at a scan rate of 120 °C·h^−1^. The experiments were carried out in 50 mM sodium phosphate buffer pH 7.4 and 50 mM glycine/HCl buffer pH 2.5. Protein concentration was typically 40 μM. Instrumental baselines were recorded before each experiment with both cells filled with buffer and subtracted from the experimental thermograms of the protein samples. Consecutive reheating runs were carried out to determine the reversibility of the thermal denaturation. The partial molar heat capacity (C_p_) was calculated from the experimental DSC thermograms using Origin software (OriginLab, Northampton, MA, USA). Reversible unfolding thermograms were fitted using two-state N ↔ U or three-state N ↔ I ↔ U unfolding models. In case of irreversible unfolding, we used a Lumry–Eyring denaturation model [30] (N ↔ U → F) to fit the experimental thermograms. The DSC thermograms measured with mixtures of covNHR proteins and CHR peptides were analyzed with a 1:1 binding model (N + L ↔ NL) coupled to the Lumry–Eyring model, as described elsewhere [23].

### 2.5. Isothermal Titration Calorimetry

Isothermal titration calorimetry (ITC) measurements were carried out in a Microcal VP-ITC calorimeter (Malvern Panalytical, Malvern, UK). The proteins were titrated with 25 injections of 5 μL peptide solution at 480 s intervals. Protein concentration in the cell was around 10 μM, while the peptide concentration in the syringe was typically 200–300 μM. The experiments were carried out in 50 mM phosphate buffer (pH 7.4) at 25 °C. The experimental thermograms were baseline corrected, and the peaks were integrated to determine the heat produced by each ligand injection. Heat of dilution of the peptides was measured in independent titrations with only buffer in the calorimeter’s cell and subtracted from the heat of the titration of the protein. Finally, each heat was normalized per mole of injected ligand. The resulting binding isotherms were fitted using a binding model of independent and equivalent sites, allowing the determination of the binding constant, K_b_; the binding enthalpy, ΔH_b_; and the binding stoichiometry, n. From these values, the standard Gibbs energy and entropy of binding could be derived as ΔG_b_ = −RT ln K_b_ and ΔS_b_ = (ΔH_b_ − ΔG_b_)/T. In some experiments, the binding model was corrected from the influence of protein self-association, as described in the Results.

### 2.6. HIV-1 Inhibition Assays

The inhibition of HIV-1 replication was determined using the conventional TZM-bl assay measured as a function of reductions in Tat-regulated Firefly luciferase (Luc) reporter gene expression [31]. The viruses used for TZM-bl cell infection were HIV SF162 strain (Tier 1, easy to neutralize subtype B strain) and CE1176 (Tier 2, difficult to neutralize subtype C strain). The IC50, the concentration (in nM) of inhibitor inducing a 50% decrease in relative luminometer units (RLU), corresponding to a 50% decrease in virus replication compared to the control, was calculated by non-linear regression using a sigmoidal Hill function, as implemented in Origin software (v.8.5, Originlab, Northampton, MA, USA).

## 3. Results

### 3.1. Structure and Stability of the Mutant Proteins

**Figure 2:**
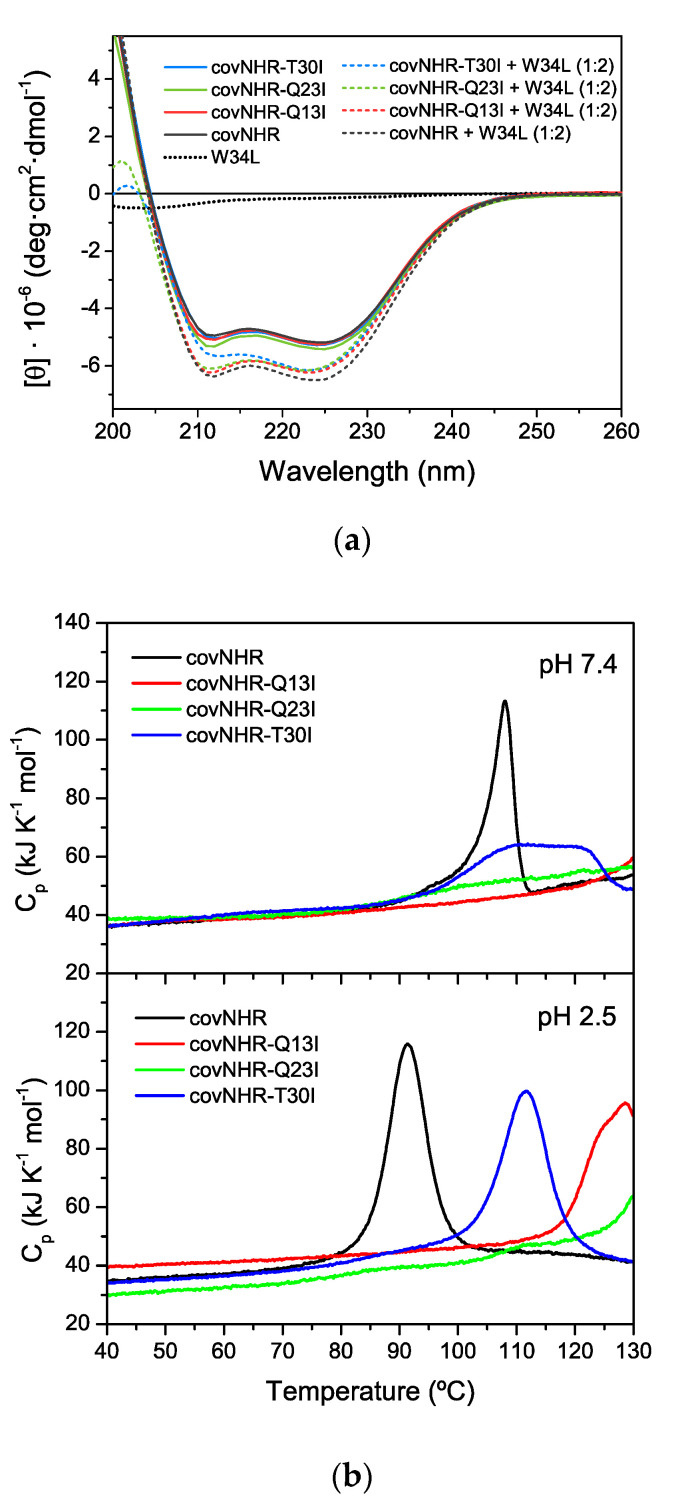
Structure and stability of the covNHR mutants. (**a**) Circular dichroism (CD) spectra of the free proteins and in complex with W34L peptide. The spectrum of the free peptide is also shown for reference. The spectra are normalized in ellipticity units per mole of protein. (**b**) Differential scanning calorimetry (DSC) thermograms of covNHR proteins at pH 2.5 and pH 7.4.

All the covNHR core mutants were expressed in *E. coli* with good yields and showed high solubility, similarly to the parent covNHR protein. CD spectra (Figure 2a) indicate highly α-helical structures with no significant differences between the four variants, indicating that the core mutations do not alter the coiled-coil structure.

To characterize the effects of the mutations on the stability of the proteins, thermal scans were monitored with CD at pH 2.5 (50 mM glycine-HCl buffer) and pH 7.4 (50 mM sodium phosphate buffer) (Figure S1). At both pH values the three mutant proteins were highly stable and the denaturation transitions fall outside the temperature range of the CD scans (up to 98 °C).

DSC scans were carried out up to 130 °C (Figure 2b). CovNHR-T30I was significantly more stable than covNHR. Strikingly, covNHR-Q13I and especially covNHR-Q23I showed extreme thermostability, with denaturation temperatures near or well above 130 °C. This indicates that the stability penalty of burying polar side chains within the coiled-coil core is larger for Gln than for Thr. At pH 2.5, the DSC reheating indicated partial reversibility of the unfolding transitions, whereas at pH 7.4 the denaturation profiles were fully irreversible. The DSC thermograms of covNHR and covNHR-T30I at acid pH could not be accurately fitted with the two-state unfolding model, but they could be fitted very well with a model of two sequential unfolding transitions (N ⇄ I ⇄ U), as described previously [22] (Figure S2). At pH 7.4 irreversible denaturation profiles were analyzed using a Lumry–Eyring model N ⇄ I → F) [23]. The thermodynamic parameters of the thermal denaturation processes are shown in Table S2. The results indicate that the Thr to Ile mutations reduce the unfolding cooperativity.

### 3.2. Binding of CHR Peptides

**Figure 3:**
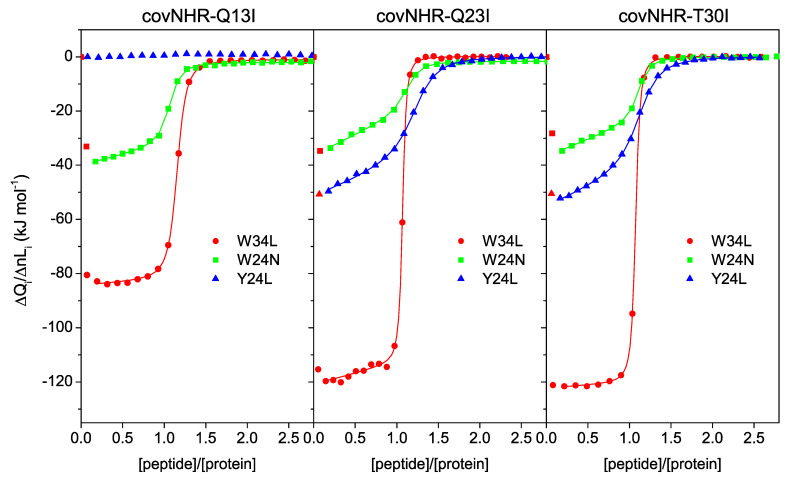
Binding of the covNHR mutants to C-terminal heptad repeat (CHR) peptides. Binding isotherms determined by isothermal titration calorimetry (ITC) for each protein to different peptides as indicated in the figure. The symbols represent the experimental heat normalized per mole of injected ligand, and the curves represent the best fittings obtained with the binding models as described in the text.

The three core mutants bind the W34L peptide (gp41 residues 117–150, more commonly known as C34), which acquires α-helical structure according to the CD spectra of the protein–peptide mixtures (Figure 2a). To elucidate the binding stoichiometry and determine the thermodynamic parameters, ITC titrations of the proteins were carried out with W34L. The binding isotherms (Figure 3) showed highly exothermic binding with very sharp sigmoidal shapes indicating high affinity and approximately 1:1 stoichiometry, similarly to that observed for the reference covNHR protein [23]. The data could be fitted using a binding model of n independent and identical sites. The binding parameters are given in Table 1. Compared to covNHR, the affinity of which for W34L was so high that it could not be assessed by ITC titration [23], the covNHR-Q13I mutant shows apparent reduction in binding enthalpy and affinity. However, the binding affinities of the Q23I and T30I variants remain very high and fall out of the upper limit of the values accessible by direct ITC titrations.

**Figure 4:**
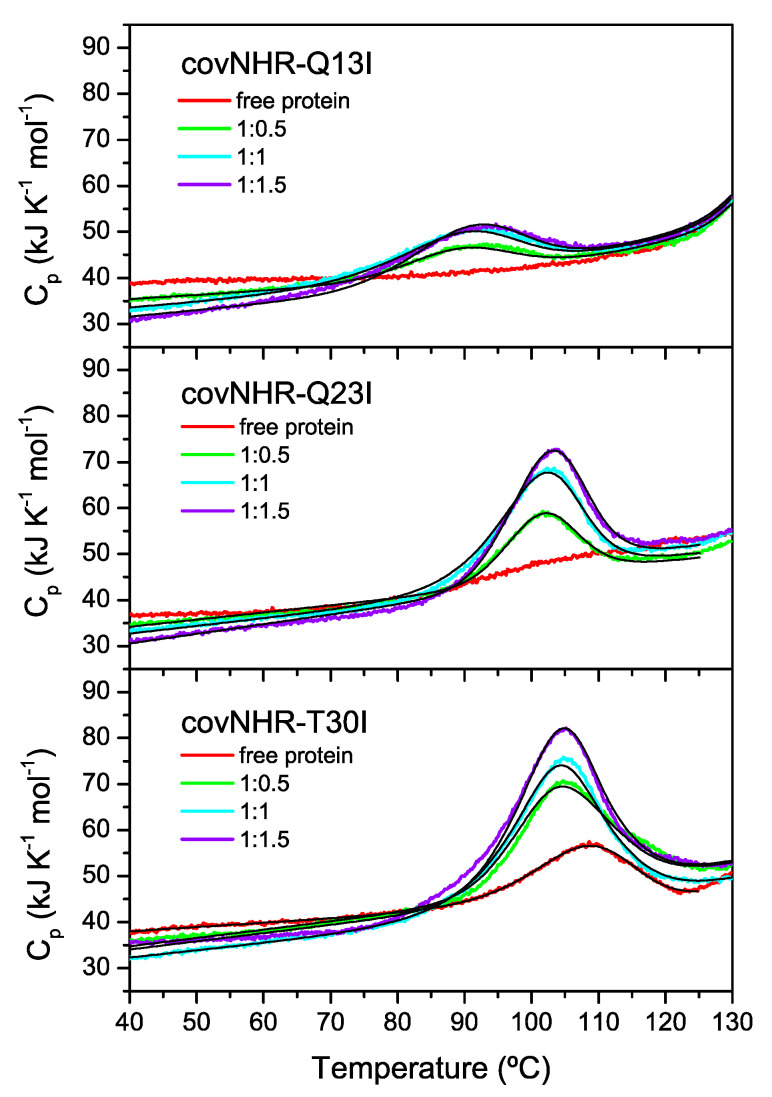
DSC analysis of thermally induced dissociation of the complexes of covNHR mutants with W34L. Protein–peptide mixtures were analyzed at the indicated molar ratios. The experimental data are represented in colors. The black solid lines represent the best fittings using the model of ligand binding coupled to a Lumry–Eyring denaturation.

Therefore, to better estimate the binding affinities, we employed an approach based on DSC, as previously used for covNHR [23], in which we analyzed the protein–peptide mixtures at different molar ratios observing the influence of the thermally induced dissociation on the DSC thermograms. Since binding is highly exothermic, dissociation is endothermic and will be forced by heating. The resulting thermograms for the three mutants are shown in Figure 4. Interestingly, due to the extreme stability of the Q13I and Q23I mutants, the peak corresponding to peptide dissociation induced by heating becomes fully separated from the denaturation transitions of the proteins, which occur at much higher temperatures. In the case of the T30I mutant, the peaks corresponding to peptide dissociation and protein denaturation partially overlap. The curves were globally fitted with a 1:1 equilibrium binding model (N + L ↔ NL) coupled to the Lumry–Eyring denaturation model (N ↔ I → F), as described previously [23]. The binding enthalpy measured by ITC at 25 °C was fixed in the fittings. The resulting binding affinities are detailed in Table 1. Strikingly, the binding constants estimated with this procedure are considerably higher than those estimated by ITC, even up to five orders of magnitude, as it is the case of the Q23I mutant. These dramatic differences are unlikely to be due to experimental errors of the calorimetric experiments, but they likely reflect some type of anomalous binding behavior that is not accounted for by the simple binding models used to analyze the data quantitatively, as discussed later in the paper.

We previously reported that the high affinity between the NHR and CHR regions is a result of cooperative contributions of several binding pockets along the NHR crevice [24]. To elucidate whether the observed changes in binding affinity produce regional effects at different pockets, we investigated the binding of shorter CHR peptides that encompass only part of the binding determinants. We carried out ITC titrations with peptide Y24L (gp41 residues 127–150), which binds to the N-terminal polar pocket (NTP) and a middle pocket (MP) of the NHR crevice, and peptide W24N (residues 117–140), which covers the MP and the deep hydrophobic pocket (HP). The binding isotherms (Figure 3) also indicate 1:1 stoichiometry for all mutants and much lower binding enthalpies than those observed for W34L, which contains the binding determinants of the three pockets. Interestingly, the Y24L peptide does not bind to the Q13I mutant, indicating that these buried glutamine side chains play an important role in the interactions at the NTP site. Despite the 1:1 stoichiometry, the ITC profiles do not match the typical sigmoidal shape expected for a simple 1:1 binding equilibrium and show a significant slope at the beginning of the titrations. These effects suggest additional processes coupled to the main binding event that alter the shape of the binding isotherm.

### 3.3. Oligomerization State

**Figure 5:**
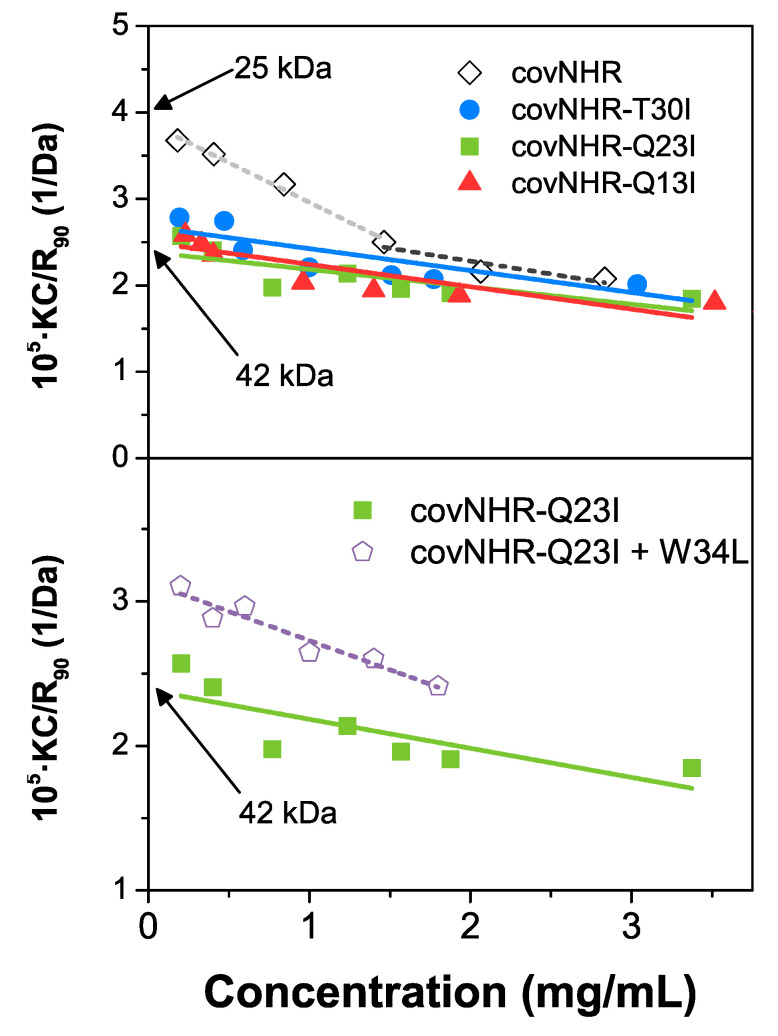
Molecular size of the covNHR proteins. Upper panel: Debye plots corresponding to the scattering intensities of protein solutions at different concentrations for the covNHR reference protein and the triple mutants. The intercepts of the linear tendencies indicate the weight averaged molar mass of the molecules. Bottom: Debye plots corresponding to the covNHR-Q23I mutant in its free form and in complex with the W34L peptide.

Since the core mutations increase the hydrophobicity of the mutant proteins, it is possible that they could also enhance their self-association propensity compared to the parent protein and this may have an influence on the binding to the peptides. To explore this possibility, we measured the apparent hydrodynamic radii (R_h_) by DLS (Table S3). The three core mutants show R_h_ values slightly larger than the covNHR reference protein, which shows a value expected for a monomer. This suggests that the mutant proteins have a propensity for self-association. To confirm this hypothesis, we measured the scattering intensities as a function of the protein concentration and made the corresponding Debye plots (Figure 5). The three mutant proteins show approximately linear tendencies with intercepts in good agreement with 1/M_w_ of the dimer (Figure 5, upper panel). In contrast, the reference protein departs at low concentration from this tendency and shows an intercept in agreement with a monomeric state. The results confirm a significantly increased self-association as dimers of the core mutants compared to the reference protein, explaining the anomalous ITC profiles observed for the core mutants. The scattering measurements with the covNHR-Q23I mutant in complex with the W34L, at a 1:2 protein/peptide ratio, show and intercept in the Debye plot corresponding to a molecular weight of about 32 kDa, closer to the expected M_w_ of the complex (25 kD) than to that of the dimer (50 kDa). These results indicate a considerable reduction in molecular mass indicating that peptide binding shifts the dimerization equilibrium towards the monomeric state, in good consistency with the 1:1 binding stoichiometry observed by ITC (Figure 3). This implies that self-association perturbs peptide binding, probably by occluding or distorting the NHR binding crevice.

To account for the influence of self-oligomerization on the ITC binding isotherms of the W24N and Y24L peptides, we fitted the curves using a model of binding coupled to a dimerization equilibrium, assuming that the peptide can only bind to the monomer. This model described very satisfactorily the ITC isotherms (Figure 4) although, due to over parameterization, the dimerization constants and the association enthalpies show very strong dependency and could not be determined independently. We found good fittings using fixed dimerization constants of 1 × 10^3^ M^−1^, corresponding to a mole fraction of dimers of about 0.5 under the conditions of the ITC experiments. Nevertheless, the peptide binding parameters have a very low dependency with the parameters of the dimerization, allowing reasonable estimates of the former from these analyses. In contrast, self-association has a very small influence on shape of the ITC isotherm for titrations with W34L and fit almost equally well to a simple 1:1 binding model.

The parameters of Table 1 indicate that peptides Y24L and W24N, which only occupy two pockets on the NHR crevice, have a much lower affinity than W34L, which covers the three pockets. The core mutations generate significant decreases in the binding enthalpies compared to the parent covNHR protein but, in general, produce relatively small changes in the binding affinity for the short peptides, except in the case of the complete abolishment of binding of Y24L to covNHR-Q13I. The loss of favorable binding enthalpy is therefore compensated by a lower entropy cost of binding, as a result of the increase in hydrophobicity produced by the mutations.

A striking result is the large discrepancy between the affinity constants of W34L derived from direct ITC experiments and the dissociation constants obtained by DSC of protein–peptide mixtures. The difference is particularly outstanding for the Q23I mutant, for which there is a difference of 5 orders of magnitude. ITC measures the heat released upon complex formation between the free protein and peptide, whereas the DSC experiments monitor the heat of dissociation of a pre-formed complex at high temperature. It is possible that these processes are not just opposite steps of the same reversible binding equilibrium. Other factors, such as the observed dimerization propensity of the proteins and the existence of kinetic limits to the binding-dissociation processes may also influence the apparent binding constants and produce inconsistent values between the two methods.

To attempt to shed light on this inconsistency, we carried out an ITC displacement experiment, in which we placed covNHR-Q13I and W34L at 1:2 molar ratio in the calorimetric cell and titrated with the reference covNHR protein from the syringe. According to the large difference in affinity and binding enthalpy, if the proteins were competing for the peptide in simple reversible binding events, the binding profile of this experiment would yield two sigmoidal steps, one at 1:0.5 covNHR/W34L stoichiometry showing the binding of covNHR to the excess free W34L peptide, and a second step at 1:1 covNHR/W34L stoichiometry corresponding to the displacement of the Q13I mutant from binding of the remaining W34L peptide, with heat corresponding to the net enthalpy balance of the processes. In contrast, the experiment did not show such profile and only a first binding step with an approximate 1:0.5 stoichiometry (Figure S3), indicating that covNHR cannot displace the Q13I mutant from the complex with W34L within the time frame of the ITC titration at 25 °C. This points to a slow dissociation of the peptide that limits the capacity of ITC to estimate these extremely high affinity constants.

### 3.4. HIV-1 Inhibition Activity

Finally, we aimed to investigate how the core mutations affected the capacity of the proteins to inhibit the HIV-1 infection in vitro. The four proteins were tested in TZM-bl neutralization assays using SF162 and CE1176 pseudoviruses (Figure S4). The IC50 values are given in Table 2. All proteins show a strong inhibitory activity in the low nanomolar range with insignificant differences between them. It is interesting that the Q13I mutant keeps a similar inhibitory potency to the other proteins, despite its considerably decreased affinity for the CHR region produced by perturbation of the interactions at the NTP pocket. The IC50 values are similar between the different proteins but much higher than the dissociation constants of the complexes with W34L derived from DSC analysis. There is no correlation between the inhibitory potency and the peptide binding affinity.

## 4. Discussion

Here we show how replacing buried polar Thr or Gln sidechains for Ile in three core positions of the covNHR protein leads to a very strong increase in thermal stability. The stability increase is much larger for Gln than Thr substitution. This is consistent with higher hydrogen bonding potential of the Gln side chain that cannot be fully satisfied in the core interior compared to the water environment. Although hydrogen bonds buried in the apolar interior are particularly strong [26], there is considerable energy penalty compared to the hydrophobic and van der Waals interactions between Ile side chains.

An immobilized water molecule was found hydrogen bonded to the Q23, Q89, and Q134 side chains in the crystallographic structure of the covNHR protein in the complex with W34L. These hydrogen bonds may add an additional enthalpic component to the stability of the covNHR protein. However, the large entropy cost of immobilizing this water molecule adds a considerable energy penalty that may explain why the Q23I variant is more stable than the Q13I one.

The Q13, Q99, and Q124 triad only establish hydrogen bonds with groups from opposite helices and do not hold any buried water molecule in the center of the NHR coiled coil. However, the Q13 side chain is implied in a hydrogen bond with a buried water molecule at the NHR–CHR interface (Figure 1), which is part of a hydrogen bond network at the NTP [23]. This has a strong implication for peptide binding as discussed below.

T30, T82, and T141 sidechains are fully buried in the crystallographic structure, but neither hydrogen bonding nor immobilized water are associated with these side chains. Thus, the increased stability observed for the Ile mutants of these residues should be ascribed to improved internal packing and enhanced hydrophobic interaction.

Lu and coworkers used a recombinant model of the SIV gp41 6HB conformation, designated N36(L6)C34, to investigate the role of the similar buried polar residues in SIV gp41 [27]. Specifically, they studied equivalent mutations to those described here, i.e., Q565I, Q575I, T582I, and T586I. They reported 15–20 °C increases in the unfolding temperatures, which are lower than the variations observed here for the Gln-Ile mutants. This difference may be caused by a lower symmetry of the core interactions of the covNHR proteins compared to the trimeric N36(L6)C34 constructs, allowing less optimal hydrogen bonding of the Gln side chains. Their Q575I variant (equivalent to covNHR-Q23I in this work) was insoluble in pure physiological buffer but soluble in presence of 1.5 M GdmHCl. Our results also show that the increased hydrophobic character produced by the mutations favors self-association of the proteins into dimeric species. These data support the hypothesis that buried polar interactions contribute to structural specificity and uniqueness at a cost of stability [28,32,33].

Nevertheless, the N36(L6)C34 constructs do not make it possible to discriminate between the effects of the mutations on the stability of the NHR trimeric coils and their influence on the interactions with the CHR region. Here we show that each set of mutations plays a different role in the NHR–CHR interaction.

Each of the triads of the mutated residues is a structural part of the regions harboring different pockets at the binding interface between covNHR and the W34L peptide (Figure 1). The Q13 triad participates in the NTP, the Q23 triad is part of the MP, and the T30 triad pertains to the HP [24]. Each set of mutations to Ile should therefore influence the conformational properties of each pocket and thereby the binding interactions with CHR peptides. The effects, however, are not restricted locally but extend to other pockets.

The strongest effects are observed for covNHR-Q13I, which shows undetectable affinity for the Y24L peptide and has a strongly decreased affinity for the W34L peptide. This is a local effect that produces also a considerable reduction in negative binding enthalpy by about one third, indicating a decrease in the favorable balance of interactions. This is justified by the disruption of the extensive network of interactions at the NTP, in which an interfacial water molecule bridges polar residues between NHR and CHR (Figure 1d). These conserved glutamine residues have a role not only in determining structural specificity of the NHR coiled coil but also in affinity and specificity of the NHR–CHR interaction. It has been shown that buried networks of hydrogen bonds in coiled coils are very important in determining the folding and oligomeric assembly in apolar environments [34] and can extend to outer shells of concentric helices, completely determining the coiled-coil topology [35].

**Figure 6:**
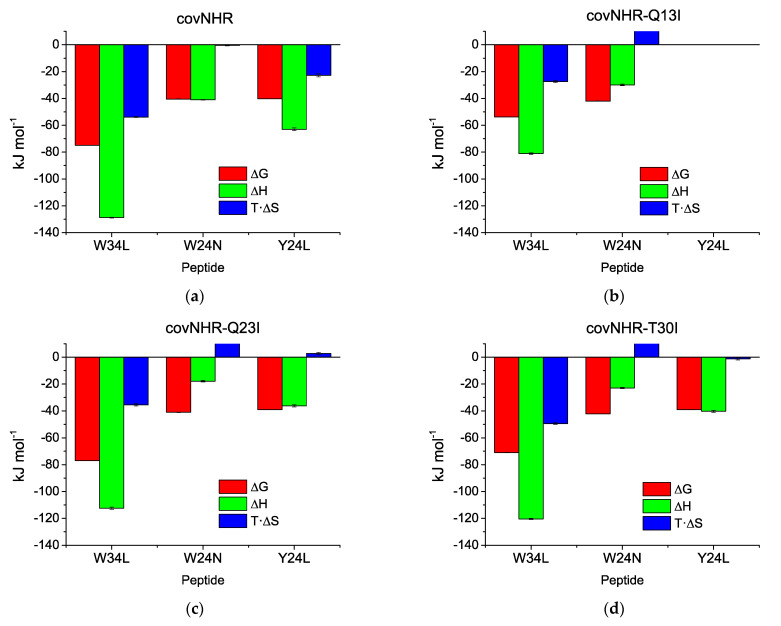
Thermodynamic signature of the binding of CHR peptides to the covNHR proteins. Enthalpy, entropy, and Gibbs energy of binding were obtained from parameters listed in Table 1. (**a**) Reference covNHR protein; (**b**) covNHR-Q13I; (**c**) covNHR-Q23I; (**d**) covNHR-T30I.

The Q23I and T30I sets of mutations slightly reduce the binding affinity for the Y24L peptide. In contrast, the three triads of polar-to-Ile mutations produce small increments in the binding affinity for the W24N peptide. All these effects are accompanied by considerable reductions in favorable binding enthalpy, which are compensated by increased entropy of binding (Figure 6), resulting in strong enthalpy–entropy compensation. Since the mutated residues do not participate directly in the binding interface, except Q13, the only mechanism that can explaining these thermodynamic changes is a strong coupling between binding and structural tightening, as previously proposed [23].

We have recently reported that the NHR coiled coil is composed of two subdomains with different stability [36]. The N-terminal half, containing the NTP and MP pockets, is intrinsically unstable, whereas the C-terminal part harboring the HP pocket shows a much higher stability. It is interesting to notice how the highly stabilizing core mutations studied here enhance binding at the C-terminal part while reducing affinity at the N-terminal part, especially the Q13I mutations. Replacement of polar residues for isoleucine appears therefore to diminish the energetic linkage between CHR binding and stability of the N-terminal subdomain. A possible reason for these observations is that the gp41 NHR sequence may be finely tuned to balance the stability of the NHR trimer of helices in order to avoid exposure of a too stable binding groove that would expose persistently conserved epitopes susceptible to neutralization.

Despite the observed effects in stability and binding affinity for the CHR target peptides, the in vitro inhibitory activity of all the protein variants is quite similar. This result is in agreement with our previous study of a mutant covNHR protein that also showed abolished binding at the NTP but enhanced binding at the HP [23], similarly to what is observed here for the Q13I mutant. We have previously shown that strong inhibition activity can be achieved with a minimum of two NHR pockets (MP + HP or MP + NTP) [23,24]. However, the inhibitory activity of the covNHR proteins is quite uncorrelated with the overall binding affinity for CHR. It has been proposed that inhibitor access to gp41 CHR is kinetically limited by a slow and transient exposure of the CHR target to interaction with external NHR binders such as 5-helix [37], so that IC50 values in inhibition assays are actually inversely correlated with the association rates [38], which are similar for different mutant proteins that have binding affinities spanning several orders of magnitude. This explains why the protein variants studied here show a similar inhibitory activity.

This and previous work [22,23,36] show that the covNHR proteins inhibit a broad variety of HIV-1 strains with high potency, which highlights their strong potential to be developed as therapeutic tools against HIV-1. In the development of a peptide- or protein-based antiviral, it is important not only to achieve high inhibitory potency but also other properties such as high solubility, ease of production and purification, high stability, and structural uniqueness. These are key aspects in drug formulation, as well as for appropriate bioavailability and pharmacokinetics. For instance, T20 (enfuvirtide) treatment requires a very high dosage due to its short half-life in the blood stream, which limits its clinical use [39]. In another example, T-1249, a promising enfuvirtide successor, was discontinued due to formulation difficulties [40]. Therefore, improving the conformational and stability properties of protein-based drug candidates is crucial. The core mutations described here produce strong stability increases without affecting significantly their high solubility and high production yield. However, the mutations enhance their propensity for self-association, which interferes with the potential to recognize their target. Additional studies in vivo will be necessary to establish whether or not the strategy of protein stabilization by mutating core polar residues results in better drug candidates against HIV-1.

## Figures and Tables

**Table 1:** Thermodynamic parameters of binding of gp41 CHR peptides to the covNHR proteins determined by isothermal titration calorimetry (ITC) or differential scanning calorimetry (DSC).

Protein Variant	Peptide	Technique	K_b_ (M^−1^)	K_d_ (nM)	ΔH_b_ (kJ·mol^−1^)	n
covNHR ^(1)^	W34L	ITC	Not measurable		−128.8 ± 0.3	1.01
		DSC	(1.32 ± 0.03) × 10^13^	0.000076 ± 0.000002		
	W24N	ITC	(1.25 ± 0.11) × 10^7^	80 ± 7	−41 ± 0.3	0.83
	Y24L	ITC	(1.11 ± 0.09) × 10^7^	90 ± 7	−63 ± 1.0	0.83
covNHR-Q13I	W34L	ITC	(4.4 ± 0.3) × 10^7^	22.7 ± 1.3	−81.1 ± 0.6	1.16
		DSC	(2.62 ± 0.05) × 10^9^	0.382 ± 0.007		
	W24N	ITC	(2.3 ± 0.3) × 10^7^	44 ± 5	−29.9 ± 0.6	1.07
	Y24L	ITC	Undetectable binding			
covNHR-Q23I	W34L	ITC	(2.0 ± 0.3) × 10^8^	5.1 ± 0.7	−112.4 ± 0.8	1.07
		DSC	(2.96 ± 0.06) × 10^13^	0.000034 ± 0.000001		
	W24N	ITC	(1.61 ± 0.23) × 10^7^	62 ± 9	−17.9 ± 0.6	1.14
	Y24L	ITC	(7.0 ± 0.5) × 10^6^	142 ± 9	−36.3 ± 0.8	1.24
covNHR-T30I	W34L	ITC	(1.58 ± 0.08) × 10^8^	6.3 ± 0.3	−120.4 ± 0.5	1.08
		DSC	(2.6 ± 0.3) × 10^12^	0.00038 ± 0.00004		
	W24N	ITC	(2.48 ± 0.24) × 10^7^	40 ± 4	−23.0 ± 0.4	1.12
	Y24L	ITC	(7.2 ± 0.4) × 10^6^	139 ± 8	−40.4 ± 0.7	1.14

^(1)^ Data from refs. [23,24].

**Table 2:** HIV-1 inhibition activity of covNHR proteins measured with the standard TZM-bl assay using SF162 (subtype B) and CE1176 (subtype C) pseudoviruses. Values correspond to IC50 in nM units.

Protein Variant	SF162	CE1176
covNHR	8.0 ± 1.3	10.8 ± 0.9
covNHR-Q13I	8.9 ± 1.3	10.7 ± 0.8
covNHR-Q23I	7.1 ± 0.9	5.3 ± 1.1
covNHR-T30I	8.6 ± 0.5	7.4 ± 0.05

## Data Availability

The experimental data presented in this study are openly available at the institutional repository of the University of Granada (Digibug) at http://hdl.handle.net/10481/67394, accessed on 11 April 2021.

## Supplementary Materials

The following are available online at https://www.mdpi.com/article/10.3390/biom11040566/s1, Figure S1: Thermal scans monitored by CD; Figure S2: Fitting of DSC thermograms; Figure S3: ITC displacement experiment; Figure S4: In vitro inhibition assays; Table S1: Amino acid sequences of the covNHR proteins; Table S2: Thermodynamic and kinetic parameters of thermal unfolding; Table S3: Apparent hydrodynamic radii of the covNHR proteins.
