# Supplementary material for: Extremely Thermostabilizing Core Mutations in Coiled-Coil Mimetic Proteins of HIV-1 gp41 Produce Diverse Effects on Target Binding but Do Not Affect Their Inhibitory Activity
Source: Biomolecules. 2021 Apr 12;11(4):566. doi: 10.3390/biom11040566 (PMC8069472; doi:10.3390/biom11040566)
Supplement: Supplementary file 1 [file biomolecules-11-00566-s001.pdf]

Supplementary Material to:

Extremely Thermostabilizing Core Mutations in Coiled-Coil Mimetic Proteins of HIV-1 gp41 Produce Diverse Effects on Target Binding but Do Not Affect Their Inhibitory Activity

Mario Cano-Muñoz [1,*], Samuele Cesaro [1,†], Bertrand Morel [1,‡], Julie Lucas [2], Christiane Moog [2] and Francisco Conejero-Lara [1,*]

[1] Departamento de Química Física, Instituto de Biotecnología, Unidad de Excelencia de Química Aplicada a Biomedicina y Medioambiente (UEQ), Facultad de Ciencias, Universidad de Granada, 18071 Granada, Spain; mariocano@ugr.es (M.C-M.), bmorel@ugr.es (B.M.); conejero@ugr.es (F.C-L.)

[2] INSERM U1109, Fédération de Médecine Translationnelle de Strasbourg (FMTS), Université de Strasbourg, Strasbourg, France; julie.lucas@etu.unistra.fr (J.L.); c.moog@unistra.fr (C.M.)

* Correspondence: mariocano@ugr.es (M.C-M.), conejero@ugr.es (F.C-L.); Tel.: +34 958242371 (F.C-L.)

† Present address: Department of Neurosciences, Biomedicine and Movement Sciences, Section of Biological Chemistry, University of Verona, Strada Le Grazie, 8, 37134, Verona, Italy; samuele.cesaro@univr.it (S.C.)

‡ Present address: Angany Innovation, 1 voie de l'innovation, Pharmaparc II, 27100 Val de Reuil, France; bmorel76@gmail.com

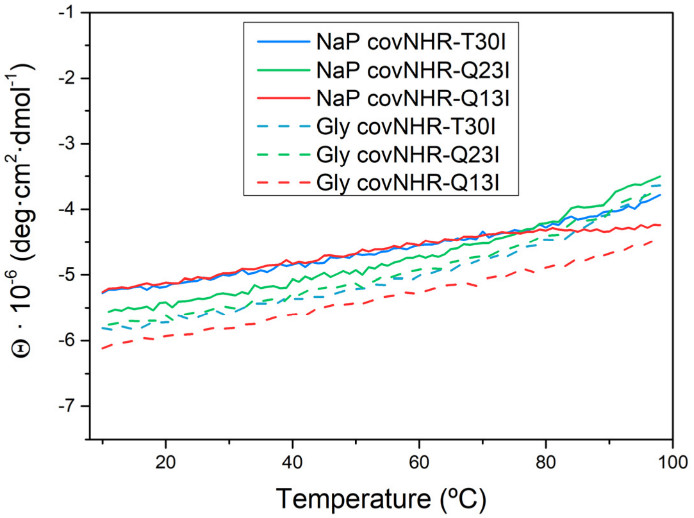

Figure S1: Thermal scans monitored by CD at 222 nm of the covNHR protein variants in 50 mM sodium phosphate buffer pH 7.4 (NaP) and 50 mM glycine/HCl buffer at pH 2.5 (Gly).

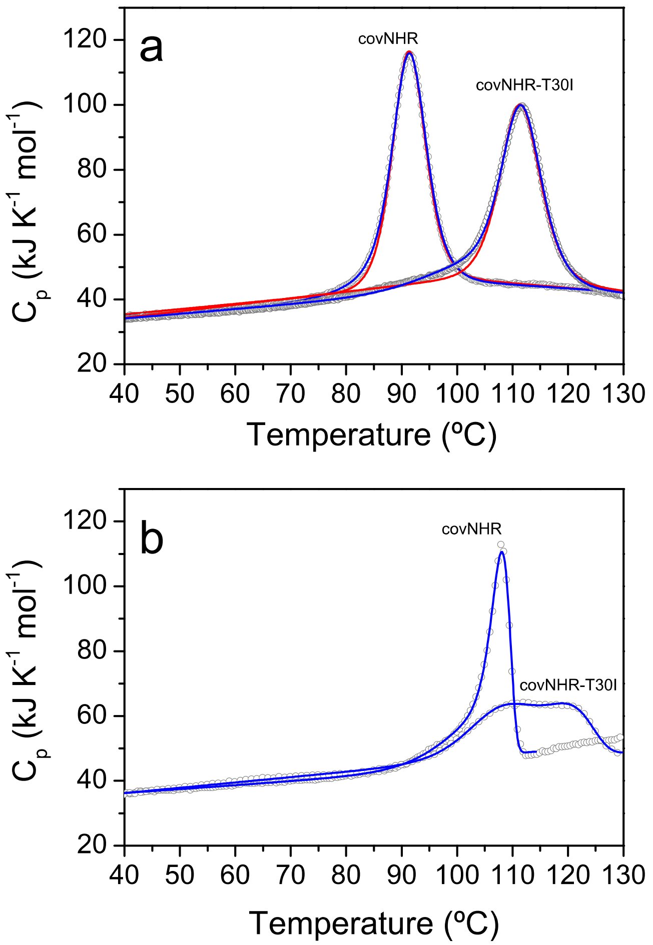

Figure S2: Fitting of DSC thermograms. DSC thermograms of covNHR and covNHR-T30I measured at pH 2.5 in 50 mM Glycine/HCl buffer (a) and pH 7.4 in 50 mM phosphate buffer (b). The experimental data are represented by symbols. The solid lines in (a) represent the best fits using a two-state unfolding model N ⇄ U (red) and a three-state sequential model N ⇄ I ⇄ U (blue). The blue solid lines in (b) represent the best fits using a Lumry-Eyring irreversible denaturation model N ⇄ I → F.

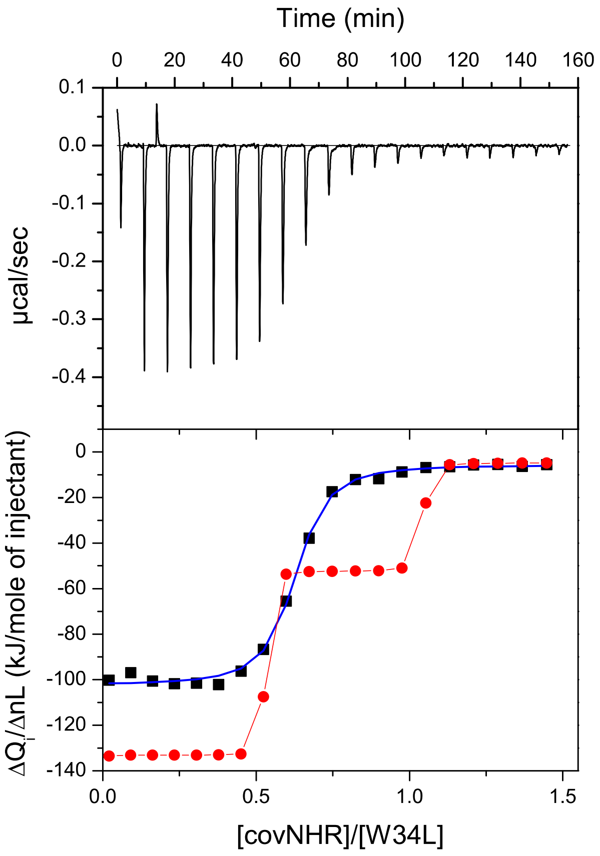

Figure S3: ITC displacement experiment in which a covNHR-Q13I:W34L mixture at a 1:2 molar ratio in the cell was titrated with the reference protein covNHR from the syringe. covNHR-Q13I was at 5 µM and W34L at 10 µM in the cell. (Upper panel) Experimental thermogram at 25°C; (lower panel) ITC isotherm showing the normalized heats per mole of injected protein. The black symbols represent the experimental heats and blue line represents the fit using a n-independent binding sites model. The predicted isotherm assuming rapid binding equilibria and using the parameters of Table 1 is represented in red.

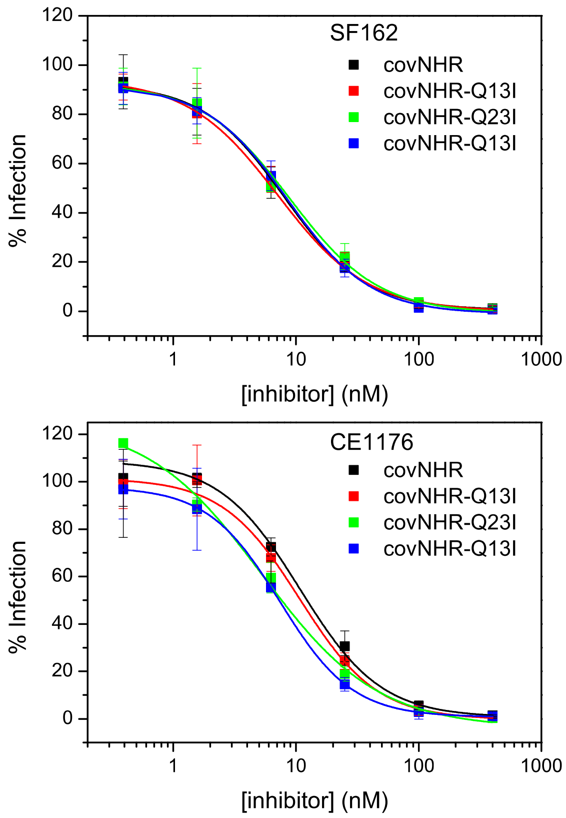

Figure S4: *In vitro* inhibition assays of infection of TZM-bl cells with SF-162 (upper panel) and CE1176 (lower panel) pseudoviruses by different concentrations of covNHR proteins. The data represent the mean values of three independent measurements, and the error bars correspond to the SDs. The solid lines show the fittings using a sigmoidal Hill function.

Table S1: Amino acid sequences of the covNHR proteins and gp41 CHR peptides studied in this work. Residues in bold correspond to the Polar-to-Isoleucine mutations. Loops connecting the helices are identical for all proteins. All constructs were expressed in E. coli with a N-terminal methionine and a C-terminal His-tag sequence GGGGSHHHHHH.

**WT gp41**	**NHR sequence 30-81**
	ARQLLSGIVQQQNNLLRAIEAQQHLLQLTVWGIKQLQARILAVERYLKDQQL
	**Helix 1**
**covNHR**	ARQELSGIVQK**Q**NNLLRQIEA**Q**QHLLQL**T**VSKIKQLQARILAVERYLKDQQL
**covNHR-Q13**	ARQELSGIVQK**I**NNLLRQIEAQQHLLQLTVSKIKQLQARILAVERYLKDQQL
**covNHR-Q23**	ARQELSGIVQK**Q**NNLLRQIEA**I**QHLLQL**T**VSKIKQLQARILAVERYLKDQQL
**covNHR-T30**	ARQELSGIVQK**Q**NNLLRQIEA**Q**QHLLQL**I**VSKIKQLQARILAVERYLKDQQL
	**Loop1**
	GKGNQ
	**Helix 2**
**covNHR**	PQQDKLYREVALIRAQLQKIESE**T**LQLLHQ**Q**AEIERELNN**Q**EQEIGSLKQR
**covNHR-Q13**	PQQDKLYREVALIRAQLQKIESETLQLLHQQAEIERELNN**I**EQEIGSLKQR
**covNHR-Q23**	PQQDKLYREVALIRAQLQKIESETLQLLHQ**I**AEIERELNNQEQEIGSLKQR
**covNHR-T30**	PQQDKLYREVALIRAQLQKIESE**I**LQLLHQQAEIERELNNQEQEIGSLKQR
	**Loop2**
	GLIDG
	**Helix 3**
**covNHR**	PLLSGIDQQ**Q**NNLKRAIEA**Q**KHLLQL**T**VWGIKQLQARILAVERYLKDQQL
**covNHR-Q13**	PLLSGIDQQ**I**NNLKRAIEAQKHLLQLTVWGIKQLQARILAVERYLKDQQL
**covNHR-Q23**	PLLSGIDQQQNNLKRAIEA**I**KHLLQLTVWGIKQLQARILAVERYLKDQQL
**covNHR-T30**	PLLSGIDQQQNNLKRAIEAQKHLLQL**I**VWGIKQLQARILAVERYLKDQQL
	**CHR peptides**
**W34L**	WMEWDREINNYTSLIHSLIEESQNQQEKNEQELL
**W34N**	WMEWDREINNYTSLIHSLIEES
**Y24L**	YTSLIHSLIEESQNQQEKNEQELL

Table S2: Thermodynamic and kinetic parameters of thermal unfolding of covNHR and covNHR-T30I derived from the fittings shown in Figure S2.

pH 2.5; model N ⇄ I ⇄ U					
**Protein**	**$\Delta H_{I\text{-}N}$ (kJ·mol^{-1})**	**$T_{m,1}$ (°C)**	**$\Delta H_{U\text{-}I}$ (kJ·mol^{-1})**	**$T_{m,2}$ (°C)**	**$\Delta C_{p,U\text{-}N}$ (kJ·K^{-1}·mol^{-1})**
covNHR	188 ± 1	89.1 ± 0.1	467 ± 1	90.41 ± 0.02	6.41 ± 0.06
covNHR-T30I	185.6 ± 0.6	102.5 ± 0.1	475.8 ± 0.7	111.4 ± 0.01	2.27 ± 0.06

pH 7.4; model N ⇄ I → F						
**Protein**	**$\Delta H_{I\text{-}N}$ (kJ·mol^{-1})**	**$T_{m,1}$ (°C)**	**$\Delta H_{F\text{-}N}$ (kJ·mol^{-1})**	**E_a [(1)] (kJ·mol^{-1})**	**T* [(2)] (°C)**	**$\Delta C_{p,F\text{-}N}$ (kJ·K^{-1}·mol^{-1})**
covNHR	237 ± 2	102.8 ± 0.2	423.0 ± 1.4	685 ± 3	107.9 ± 0.01	3.85 ± 0.13
covNHR-T30I	269.8 ± 0.7	109.25 ± 0.05	408 ± 2	270 ± 3	125.6 ± 0.09	-2.34 ± 0.13

(1) Arrhenius activation energy for the irreversible denaturation process.
(2) Temperature at which the rate of denaturation, k_F, is equal to 1 min^{-1}.

Table S3: Apparent hydrodynamic radii of the covNHR proteins measured by dynamic light scattering at a protein concentration of 20 μM.

**Protein**	**R_h (nm)**
covNHR	2.7
covNHR-Q13I	3.0
covNHR-Q23I	2.8
covNHR-T30I	3.0
covNHR-Q23I + W34L (1:2)	2.7
